# Being tested but not educated – a qualitative focus group study exploring patients’ perceptions of diabetic dietary advice

**DOI:** 10.1186/s12875-018-0892-5

**Published:** 2019-01-03

**Authors:** Maria Alonso Arana, Jose Maria Valderas, Josie Solomon

**Affiliations:** 1 Health Services and Policy Research, University of Exeter Medical School, Exeter, UK; 2 Faculty of Health and Life Sciences, De Montfort University, Leicester, UK

**Keywords:** Diabetes, Type 2 diabetes, Diet, Dietary advice, Patients’ perspectives, Patient education, Qualitative study

## Abstract

**Background:**

Diet is a key component of the management of diabetes. Several studies suggest that patients receive insufficient and inadequate information. As a first step for developing an intervention for improving dietary advice in primary care, we aimed to explore patients’ experience of receiving dietary advice; their attitudes towards that advice; their perceived dietary advice needs, and any barriers faced in adopting a diet that supports the management of their diabetes.

**Methods:**

A qualitative study with three focus groups (20 purposively sampled participants) was conducted with adult primary care patients with Type 2 diabetes in 2016. A semi-structured topic guide was developed from the literature. The focus groups were audio recorded and transcribed. The data were analysed by emergent themes analysis. Data saturation was achieved in the third focus group.

**Results:**

The majority of participants were given dietary advice in the form of a generic healthy eating leaflet from a Practice Nurse. Participants had their Haemoglobin A1c (HbA1c) reviewed regularly, but the results seemed not to be linked with review of dietary habits. The test was perceived as being a “pass or fail”, judgmental experience. Participants felt tested but not educated.

**Conclusion:**

Individuals with type 2 diabetes seem not to receive dietary advice according to their expectations. Information collected as part of the study can be used to inform the development of interventions aimed at improving dietary advice in this population.

## Background

Type 2 diabetes is a major health concern worldwide. It is estimated that 8.5% of the world’s adult population was diabetic in 2014, of which 90% were type 2 diabetics [1]. In the United Kingdom (UK), the prevalence of diabetes was over 3.8 million people in 2015 and is estimated to rise to 5 million by 2025 [2, 3]. The direct cost to the National Health Service (NHS) in the UK is £9.8 billion, 10% of the total NHS budget [4]. The long-term complications of type 2 diabetes are well documented, with the most common one, cardiovascular disease, causing 80% of deaths [5].

However, the condition can be reversed. A recent trial found that 9 out of 10 people who followed a diet and lost over 15 kg put their type 2 diabetes into remission [6]. Effective dietary advice therefore plays a crucial role in the treatment of diabetes.

Current National Institute for Health and Care Excellence (NICE) Guidelines advise that patients should receive structured nutritional education when they are first diagnosed, and this should be reinforced annually. Nutritional education should be provided in a personalised way, tailored to the patient’s particular needs and preferences and should be provided by qualified professionals [7].

Despite these clear recommendations, in the UK, a study [8], found that patients received little or no information on what to eat during the initial weeks or months following diagnosis and as a result made adverse changes in their diet. UK data on prevalence of dietary advice in primary care is lacking. However, an Australian study, which provides an estimate for a partially analogous system, found that only 43% of patients report to have received dietary advice from their general practitioners (GPs) [9].

Even when dietary advice is given, patients report dissatisfaction with what they perceive as generic lifestyle counselling [10], a lack of individual care, and confusion from content of advice and conflicting dietary messages [11–14].

Previous studies have focused either on newly diagnosed type 2 diabetics or advice from specific professional groups as part of a larger study on diabetes. This study focuses solely on perceptions of dietary advice from any healthcare professional, for patients with varying lengths of type 2 diabetes. The study aims to explore patients’ experience of the dietary advice they received from Healthcare Professionals in Primary Care; their attitudes towards that advice; their perceived dietary advice needs, and any barriers faced in adopting a diet that supports the management of their diabetes.

## Methods

An interpretive qualitative focus group study was conducted in primary care in England between January and February 2016. A purposive sampling strategy [15] was used to recruit individuals with type 2 diabetes from three GP practices, three community pharmacies, a dental practice, a local community centre and a local Diabetes UK group. This strategy was used to maximize diversity in terms of demographic characteristics, time since diagnosis, type of treatment and degree of engagement with healthcare.

Participants were approached through flyers with a reply slip. These flyers were worded in a neutral manner and were directed to all type 2 diabetic patients, who might be interested in discussing diet. No distinction was made in the recruitment, between those who were or were not satisfied with any previous dietary advice that they had received. On receipt of the reply slip, participants were contacted by the researcher to confirm eligibility (adults over the age of 18 with a diagnosis by a doctor of type 2 diabetes), and to explain the aims of the study. Potential participants were provided with a participant information sheet. If participants were willing to participate they were asked to sign a consent form and were subsequently invited to attend one of the focus groups. One individual declined the invitation to participate.

A semi-structured topic guide was used in the focus groups, which had been developed from the literature review. The discussions started with a brief introduction of each participant about their diabetes, treatment and its duration. Subsequently, open questions assessed participants’ knowledge on healthy eating and their perception of the importance of the advised diet in the overall management of their diabetes. Follow-up questions were asked afterwards referring to the quality of the healthy eating advice received in primary care.

Three focus groups were conducted in January and February 2016, in a private meeting room. Each focus group lasted for one hour. The groups were co-facilitated by an experienced pharmacist (MAA) and one or two other healthcare professionals, who were another pharmacist, a dentist and a retired nurse. The groups were audio recorded, with participants having confirmed agreement for recording. These recordings were then transcribed verbatim and each participant was assigned a participant identity code. The data were analysed manually by emergent themes analysis [16] by two of the authors (MAA, JS). A sample of the data were initially analysed independently by MAA and JS and then compared, leading to development of the coding tree. Data saturation was achieved in the third focus group.

## Results

The total sample consisted of 20 participants, 11 female and 9 male, aged between 40 and 89 years, across three focus groups. Some participants had been recently diagnosed, whilst others had been managing their conditions for up to 26 years. Treatment plans ranged from diet and lifestyle management only (45%), to oral antidiabetic drugs (40%), and insulin (15%) (See Table 1).

**Table 1:** Participant characteristics

Participant	Gender	Age-ranges (years)	Diabetes duration (years)	Type of treatment
P1G1	Male	60–69	7	Diet only
P2G1	Female	60–69	3	Diet only
P3G1	Female	80–89	20	Insulin
P4G1	Female	50–59	1 ½	Diet only
P5G1	Female	50–59	3	Oral treatment
P6G1	Female	60–69	2 ½	Diet only
P7G1	Female	80–89	5	Diet only
P8G1	Female	70–79	2	Diet only
P1G2	Female	70–79	4	Diet only
P2G2	Female	70–79	6	Diet only
P3G2	Male	60–69	12	Insulin
P4G2	Male	70–79	6	Oral treatment
P5G2	Female	80–89	2	Oral treatment
P6G2	Female	80–89	10	Oral treatment
P7G2	Male	70–79	26	Insulin
P1G3	Male	70–79	15	Oral treatment
P2G3	Male	70–79	6	Oral treatment
P3G3	Male	40–49	10	Oral treatment
P4G3	Male	70–79	Newly diagnosed	Diet only
P5G3	Male	80–89	10	Oral treatment

Five over-arching themes were identified relating to patients’ expectations for dietary advice: mode of delivery, content, interaction with healthcare professionals, blood sugar testing, and feasibility of adapting it to everyday life.

### Mode of delivery

Some patients did not receive any advice, and for others it was not timely, as there was a long delay between diagnosis and receiving advice.

*“Well no I can’t say the General Practice seems to be good, but, no, I have had practically no advice from them actually from the start.” P6G2*

For those that did receive advice, this consisted mostly of a one-off provision of leaflets, which were generic, often for cardiovascular disease not diabetes (for example booklets of “Eating Well” by the British Heart Foundation [17]), sometimes out-of-date or not available when requested. All patients seemed dissatisfied with this type of advice, as they regarded it as unhelpful.

*“And then the practice nurse gave me a booklet on healthy eating for heart disease and said it’s the same sort of thing but actually it’s not.” “The nurse says we haven’t got any diabetes ones so you can have a heart one, it’s the same.” P4G1*

*“From the GP surgery all I have had is an “Eat Well Plate” thing that was out of date anyway. And I followed the advice they gave me when I was first diagnosed and it didn’t work for me anyway.” P1G1*

Patients desired one-to-one dietary advice, especially those who were on “diet only” as a treatment, and who had been diagnosed more recently. Patients felt that they needed to have a “genuine conversation” with their health providers about diet, rather than a generic leaflet on diet.

A small number of patients were referred to local group sessions for dietary advice. Group sessions were seen as more helpful than leaflets, but they were not tailored to patients’ needs, they used the “Eat Well Plate”, were focused mostly on portion control, and took months to get an appointment. Financial cuts caused the cessation of the local group sessions. Most patients felt that regular support group meetings would be beneficial. Some patients had therefore found their own information and support by contacting diabetic or private weight loss organizations.

### Content

The generic eating advice tended to recommend savoury carbohydrates and fruit, which caused confusion for many participants: which types of carbohydrates and sugars, does it matter how food is cooked, and how much is a portion? As a consequence some participants latched on to certain “food rules” without understanding the rationale.

“*I hadn’t eaten bacon for five years, and he said ‘there is no reason why you shouldn’t eat bacon’, but the information I got first said things like bacon and sausages are processed meat, you don’t want to have too much of it all.”* P3G3

Participants were also confused by contradictory dietary messages received from various healthcare professionals and changes in advice over time.

### Interaction with healthcare professionals

The majority of dietary advice was from Practice Nurses, with very occasional advice from a GP or dietitian. Dietary advice was not included in the six-monthly reviews with Practice Nurses. Patients doubted that Practice Nurses were adequately trained in nutrition. This perception was exacerbated by some nurses who read out advice to patients “textbook style”. One participant was put off by an “un-charming, dictator style” nurse.

*“Perhaps more training is needed on this front with diabetic specialist nurses and dieticians, they need to be given more instruction from their mentors or whoever as to what they should and should not be telling people.” P3G2*

Rigid “ten minute rule” appointments were not seen as sufficient to allow patients to discuss their diet. Patients desired consultations with healthcare professionals where they could discuss their particular requirements one-to-one and not feel pressured by the time limit.

### Blood sugar testing

Participants had regular diabetic reviews every six or twelve months. The cholesterol and Haemoglobin A1C (HbA1c) results were of particular interest to patients, who wanted to know their “numbers”, but were often confused about what a particular number might mean. The numbers seemed detached from their diet and patients wanted an explanation of how they should modify their diet in light of the numbers. Participants clearly felt that their “blood sugar test” was more than a clinical test; they were being tested and judged on their behaviour. In two of the three focus groups participants spontaneously made “school” analogies.

“*If my figures are ok, she will say fine thank you, good bye basically. But I am happy with that because I have passed the test so to speak, so I can go on to the next one. It’s like going back to school.*” P4G2

“*If she gave me a number I wouldn’t really know what it meant, was it good, bad or indifferent. As long as she is happy with what I am doing I suppose I am happy with what I am doing. But I know I could do better. It sounds like my old school report, ‘could do better’*.” P6G1

“*You either pass or fail, and if you fail you get the wagging finger.”* P3G2

Some patients were satisfied with their regular reviews, but still not with their dietary advice.

There was a perception of cost cutting with some reviews having been reduced from six-monthly to annually. Also, blood glucose meters and strips were not available on prescription, which prevented patients from being aware of the impact of their diet on their condition. Many participants felt that their GP surgeries were driven by their bonus instead of their patients’ needs, and they compared it to Scotland and Wales, where they thought that more money was spent per patient.

### Application to life

Participants felt that knowing about food types and portions was not sufficient to enable them to make effective changes to their diets. They wanted to know more about interpreting food labels. Many had learnt from external sources about the dangers of low fat products being high in sugars.

“*You can always tell a diabetic when they are peering at labels.*” P8G1

Participants also wanted advice on how to combine foods together as menus and how to modify their existing diet rather than starting from scratch. They found this easier if they were already used to dietary modifications, for example being vegetarian. Many participants found it a challenge to create a varied diet and struggled with the costs of healthy eating.

“*But I find it hard to pick an interesting menu all the time, it’s very hard shopping so I do keep to certain items whether they are all ok or not I don’t know.”* P4G2

“*What I found as well is some of the things they recommend you eat are above your budget, things like you can eat are beyond your reach sometimes.”* P3G3

Another challenge was fitting in diabetic dietary requirements with other co-morbidities, for example requiring low fibre for Irritable Bowel Syndrome. Preparing healthy food takes time and energy, which was a problem for one participant with fatigue caused by anaemia and another who was not able to stand for long periods of time.

Older patients seemed to be the most reluctant to change long-standing dietary patterns that they enjoy and could feel it is too late to change habits even if that meant dying early. Many patients felt they were allowed to deviate from their diet when they were on holiday or during a festivity. They perceived they were entitled to have some treats from time to time, and that healthcare professionals allow them to do so.

*“But because like P4G3 I just adore beer I have to really fight hard just to buy a half with a meal rather than a pint, very occasionally I will have a pint.”* P1G3

Some motivations to modify diet were the desire to avoid long-term conditions; fear of long-term consequences and dislike of taking more medicines or being put on insulin.

## Discussion

For the majority of participants dietary advice was given in the form of a generic healthy eating leaflet by a Practice Nurse. Although participants had their HbA1c reviewed regularly, the results seemed to not be linked to a review of dietary habits. Participants felt tested but not educated.

Participants’ preference was for an individualised approach and a genuine conversation with a healthcare professional, and went as far as to identify the following issues: basic food categories, types of carbohydrates, portion size, recognition of the cost of healthy food for the individual, discussion about compatibility with co-morbidities, interpreting food labels, meal and menu planning and a link between their “blood sugar results” and their diet so that they could modify diet as required.

These patient requests are in line with current guidelines [7], however this study shows that they seem not to be implemented in practice.

### Comparison with previous literature

This study confirms findings from previous research showing that most diabetic patients do not receive dietary advice that is tailored to them as an individual [11, 12, 14]. This study adds more detail about the type of advice that patients received during consultations and the type of dietary education that patients feel they need to enable them to make lasting improvements to their diet. It is recognised that dietary advice sits within a context of wider lifestyle advice for cardiovascular disease and weight management. However, patients reported a need for specific advice about management of blood sugar levels through dietary control within an overall provision of holistic care.

A previous study [9] found that patients were dissatisfied with the lack or delay in dietary advice straight after being diagnosed. This study included patients with different durations of diabetes, and it showed that they did not receive on-going nutritional care throughout their treatment. Because guidelines [7] recommend providing on-going care, it was relevant to include patients with a variety of duration since diagnosis.

There have been very few in-depth studies in the United Kingdom exploring patients’ views on nutritional advice requirements in primary care. Therefore, this study has added valuable knowledge to this area.

### Strengths and limitations

The use of qualitative methods enabled the in-depth exploration of patients’ perceptions and experiences, in order to identify the types of dietary advice that they feel they need to enable and empower them to make lasting improvements to their diets and control of their diabetes. The study included participants with varying durations of diabetes, and the setting in a non-healthcare location allowed access for those who are less engaged with healthcare systems.

Limitations include the relatively low number of participants, which may affect the generalisability of the findings*.* However, views were consistent across all the focus groups and saturation of themes was achieved in the third focus group. The study was limited to one geographical location due to the self-funded nature of the study. However, flyers were available for people to collect in diverse local settings in order to obtain a maximum demographic diversity, and the study was promoted on the *Diabetes UK* website for two months; therefore, people outside the location could also attend. A basic level of literacy was required to understand the flyer and participant information sheets and therefore this may have excluded patients with poorer literacy, who may have had different views about the adequacy of dietary advice.

### Implications for policy, research and practice

Standard practice for the delivery of dietary advice for type 2 diabetics should be re-considered. Crucially dietary information should be delivered in an educational manner that genuinely enables patients to apply it to their lives. This study has identified patient requests for both the content of dietary advice and the manner in which advice is given. Further work would be needed to explore the logistics of delivering advice within specific contexts from the perspectives of healthcare professionals.

**Fig. 1:**
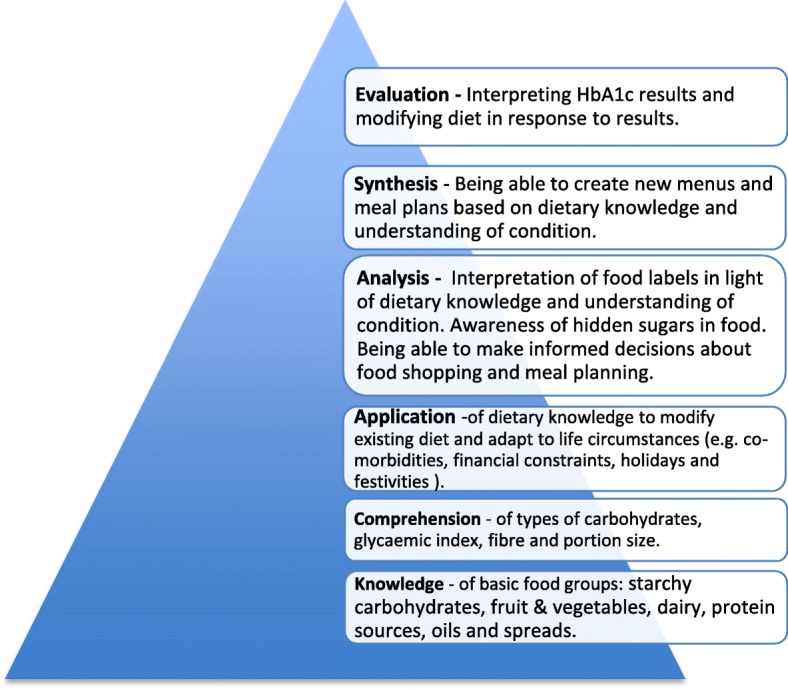
Application of Bloom’s Taxonomy (1956) to dietary education for patients with type 2 diabetes

As for research implications, consideration of the themes identified in the analysis was found to be compatible with Bloom’s work [18] and this may be worth exploring as a possible educational framework for patients. Bloom developed a taxonomy of learning, which describes a pyramid of cognitive functions moving up a scale from lower to higher order skills. This study identified a range of skills required by patients to make genuine changes to their diets, which can be mapped onto Bloom’s Taxonomy – Fig. 1.

Ideally this individualised educational approach would be delivered within the context of a genuine conversation with a nutritionally trained professional, with sufficient time.

## Conclusions

Effective dietary advice plays a crucial role in the treatment of diabetes. However, the extent and quality of dietary advice given to individuals with type 2 diabetes, seems to fall short of the standards set in guidelines, and does not meet the requirements of patients. Participants were having their HbA1c reviewed regularly, but the results seemed to not be linked to a review of dietary habits. The test was perceived as being a “pass or fail”, judgmental experience. Participants felt tested but not educated.

Dietary advice tended to consist of a generic leaflet on healthy eating and was not tailored to diabetes. Participants expressed a preference for an indvidualised approach and a genuine conversation with a healthcare professional to enable them to understand food types, make appropriate changes to their diets that fitted with their life circumstances, and to be able to adjust diet in response to changes in HbA1c levels.

This information on patients’ views about the nature of dietary knowledge and skills that would empower individuals to make improvements to their self-management of diabetes could be used to inform the development of interventions aimed at improving dietary advice in this population.

## Abbreviations

GP: General practitioner
HbA1c: Haemoglobin A1c
NHS: National Health Service
NICE: National Institute for Health and Care Excellence
UK: United Kingdom
